# Clinical utility of serial plasma cell-free DNA metagenomic next-generation sequencing assays

**DOI:** 10.1017/ice.2025.10390

**Published:** 2026-03

**Authors:** Ishminder Kaur, Bennett Shaw, Ashrit Multani, Sanchi Malhotra, Huan Vinh Dong, Christy Lukose, Kavitha Prabaker, Tawny Saleh, Young Bo Sim, Christopher N. Tymchuk, Daniel Z. Uslan, Helen Zhou, Timothy F. Brewer, Shangxin Yang

**Affiliations:** 1 Division of Infectious Diseases, Department of Pediatrics, David Geffen School of Medicine, University of California, Los Angeles, CA, USA; 2 Department of Medicine, University of California, Los Angeles, CA, USA; 3 Division of Infectious Diseases, Department of Medicine, David Geffen School of Medicine, University of California, Los Angeles, CA, USA; 4 Department of Pediatrics, David Geffen School of Medicine, University of California, Los Angeles, CA, USA; 5 Department of Epidemiology, Fielding School of Public Health, University of California, Los Angeles, CA, USA; 6 Department of Pathology and Laboratory Medicine, University of California, Los Angeles, CA, USA

**Keywords:** Plasma cell-free DNA, metagenomic next-generation sequencing, diagnostic stewardship, serial testing, clinical utility, Karius test

## Abstract

This single center retrospective observational study of serial plasma metagenomic next-generation sequencing testing shows that >95% of serial testing was without meaningful clinical impact. Only 5/173 cases were adjudicated as having significant clinical impact.

## Background

The utility of quantitative molecular burden generated by plasma cell-free DNA metagenomic next-generation sequencing assays (cf-mNGS) to monitor response to therapy has garnered attention in recent years.^1^ Serial testing is frequently utilized in infectious diseases management to monitor response to therapy, including trending viral load or repeating blood cultures to demonstrate clearance. However, there are no published data on the utility of serial plasma cf-mNGS testing to monitor response to therapy.

This study analyzes the rationale and clinical impact of serial plasma cf-mNGS at a single institution.

## Methods

### Study design

This study stemmed from a previously published retrospective study of plasma cf-mNGS testing among adult and pediatric patients at a large academic medical center in Los Angeles, California.^2^ Plasma cf-mNGS testing in our cohort was performed by Karius Inc. (Redwood City, California). The original study examined unique episodes of plasma cf-mNGS testing. Multiple plasma cf-mNGS tests performed on the same patient were included in the original study if they were separated by at least 6 months to minimize misclassification of ongoing illness as separate episodes. This study analyzes the rationale for repeat testing within 6 months of an index plasma cf-mNGS test and the clinical impact of serial plasma cf-mNGS testing by retrospective review. Because the serial test cohort was nested within the timeframe of our original study, no new stewardship interventions stemming from the original study had been introduced during the study period.

Clinical cases (test episodes) were assigned for retrospective chart review and clinical adjudication to three panels of investigators, with approximately 70 cases per panel, utilizing clinical impact definitions of the original study.^2^ Repeat tests performed for a new clinical illness were excluded from serial testing adjudication. New clinical illness was defined as an episode of illness arising after completion of therapy for index illness and/or resolution of symptoms attributable to index illness.

Quality control of clinical adjudication was performed by dedicated chart reviews by three ID specialists (T.B., A.M., and C.T.) to ensure data validity.

## Results

**Figure 1.**
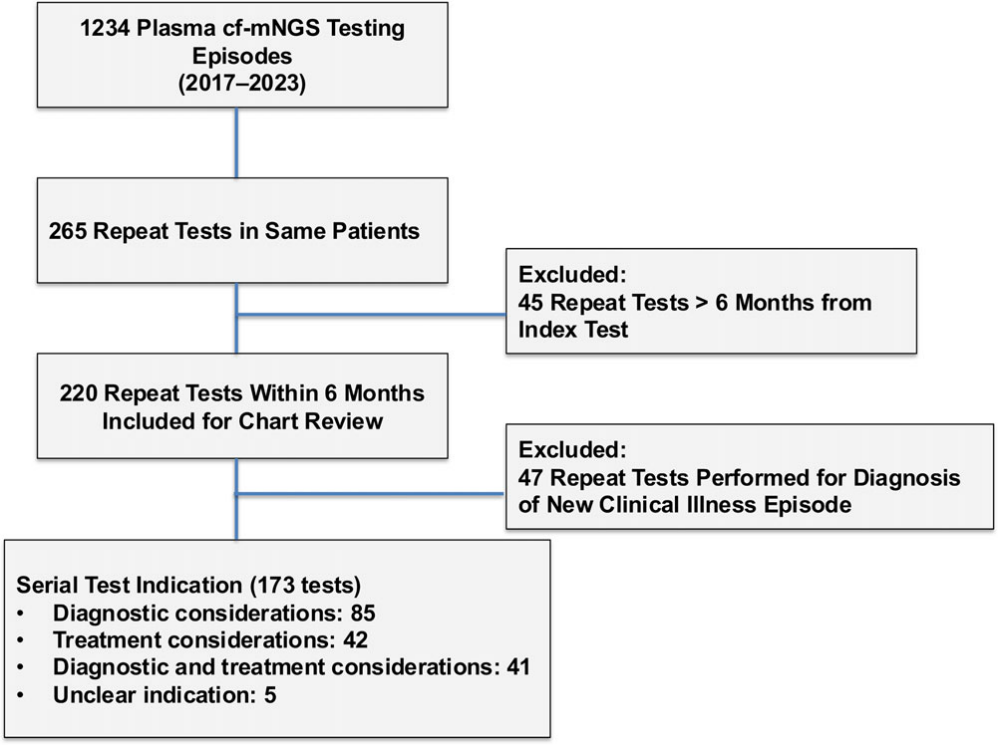
Flow diagram of included plasma cell-free DNA metagenomic next-generation sequencing assays and their indications.

We identified 1,234 plasma cf-mNGS testing episodes performed in 964 patients between January 1, 2017 and September 1, 2023. Of these, 265 tests (21%) were identified as multiple plasma cf-mNGS tests performed in the same patient. Two hundred-twenty tests (83%) were performed within 6 months of an index test in 133 patients and were included for review (Figure 1). Two-thirds of repeat tests were sent within 6 weeks of the index test (Supplementary Figure 1). The median age of patients at the time of repeat test was 14 years (range, 0–88). The median interval from index test to repeat was 21 days (range, 1–182). Most repeat plasma cf-mNGS testing was performed for diagnostic considerations (132/220, 60%), followed by treatment considerations (42/220, 20%), and a combination of treatment and diagnostic considerations (41/220, 19%).

When repeat plasma cf-mNGS testing was performed for diagnostic considerations alone (n = 132), the provider rationale included new clinical illness for the patient (47/132, 36%), clinical status change during the same illness (58/132, 44%), or provider mistrust of initial plasma cf-mNGS testing results (12/132, 9%); with the remaining 15 tests without a clearly discernable rationale. New episodes of clinical illness were excluded from further clinical adjudication as described in methods. Clinical adjudication of the remaining 85 repeat plasma cf-mNGS testing performed during the same illness revealed that majority of test results did not change management (81/85, 95%). Repeat testing demonstrated previously known organisms, no organism or no conclusive pathogen. Repeat plasma cf-mNGS testing in three immunocompromised patients with pneumonia performed following clinical status change during the same illness (time intervals of 9, 12, and 35 days from index test) revealed new diagnoses. Aspergillosis was detected in two patients by repeat plasma cf-mNGS testing (one with concurrent positive bronchoalveolar lavage galactomannan), and mucormycosis in the third patient.

Repeat plasma cf-mNGS testing performed for treatment considerations (n = 42) included monitoring pathogen molecular burden (26/42, 62%), followed by using test results in supporting antimicrobial therapy duration decisions (8/42, 19%). Clinical adjudication determined that repeat plasma cf-mNGS testing performed for treatment considerations alone resulted in meaningful clinical impact in only 2 cases (2/42, 5%), were indeterminate in 6 (6/42, 14%), and resulted in no meaningful management change in 34 cases (34/42, 81%). The 2 cases where plasma cf-mNGS testing was helpful in lending support to antibiotic decision making belonged to the same patient with *Helicobacter bilis* detected on plasma cf-mNGS testing alone (repeat tests done at 2 and 4 months); however, therapy was ultimately stopped despite ongoing pathogen detection by plasma cf-mNGS testing. Repeat plasma cf-mNGS testing done for both treatment and diagnostic considerations (n = 41) was without clinical impact in 39 cases and indeterminate in 2.

## Discussion

Approximately 20% of all plasma cf-mNGS tests performed at our institution represented repeat testing in the same patients, most often within 6 weeks of an index test. The rationale for serial testing included trending molecular microbial burden, supporting antimicrobial therapy duration decisions, and reassessing microbial etiology in the setting of clinical status change during the same illness episode. We found that serial plasma cf-mNGS testing had no meaningful clinical impact in >95% of the cases. These findings parallel our original study where majority (80%) of plasma cf-mNGS testing had no clinical impact and demonstrate the futility of serial plasma cf-mNGS testing in patients for treatment or diagnostic considerations.

Our study confirms that using the molecular burden generated by plasma cf-mNGS testing for serial monitoring of patients, even among complex cases, does not result in clinical impact in most cases. Importantly, molecular burden may not reflect clinical response and can be significantly influenced by technical variations such as sample collection, processing and NGS chemistries.^3–5^ In practice, serial results appeared more likely to reinforce clinicians’ pre-test expectations than to meaningfully inform management.

Serial plasma cf-mNGS testing did not consistently inform antimicrobial duration decisions in our cohort. Treatment courses were guided by disease process rather than by persistence or clearance of cf-mNGS-detected pathogens. For example, patients with necrotizing pneumonia appropriately received six weeks of therapy despite intermittent pathogen detection, and therapy in the *H. bilis* case was discontinued after several months despite ongoing cf-mNGS positivity. Thus, serial cf-mNGS results did not provide actionable guidance for treatment completion.

There is considerable interest in the performance of plasma cf-mNGS assays as a quantitative test similar to quantitative PCR (qPCR) assays for DNA viruses.^6–8^ Although small studies suggest that plasma cf-mNGS quantitative values may reflect bacterial burden in sepsis, differ by severity of staphylococcal device-associated endocarditis, and are higher for clinically relevant than irrelevant organisms,^9–11^ evidence remains preliminary. While our study was not designed to evaluate quantitative cf-mNGS values, their use in serial testing to trend molecular burden did not meaningfully inform clinical decision-making.

Collectively, our findings highlight the narrow scope of serial plasma cf-mNGS testing and reinforce the importance of diagnostic stewardship to optimize test utilization. Strengths of our study include the sample size and adjudication of serial tests done for the same clinical illness. Limitations include the retrospective design, non-standardized intervals between repeat testing and multiple adjudicators, though discrepancies were resolved through data validation.

Building on these findings, we are designing diagnostic stewardship interventions, including computerized decision support, ordering restrictions based on provider service, test indication, and timing from prior testing, and real-time feedback to ordering clinicians, to promote more appropriate use of plasma cf-mNGS assays. Prospective evaluation will be needed to assess their impact on reducing unnecessary plasma cf-mNGS testing.

## Supporting information

Kaur et al. supplementary material Kaur et al. supplementary material

## Supplementary material

To view supplementary material for this article, please visit https://doi.org/10.1017/ice.2025.10390
