# Supplementary figures and images for: Clinical utility of serial plasma cell-free DNA metagenomic next-generation sequencing assays
Source: Infect Control Hosp Epidemiol. 2026 Jan 13;47(3):325–7. doi: 10.1017/ice.2025.10390 (PMC12932917; doi:10.1017/ice.2025.10390)

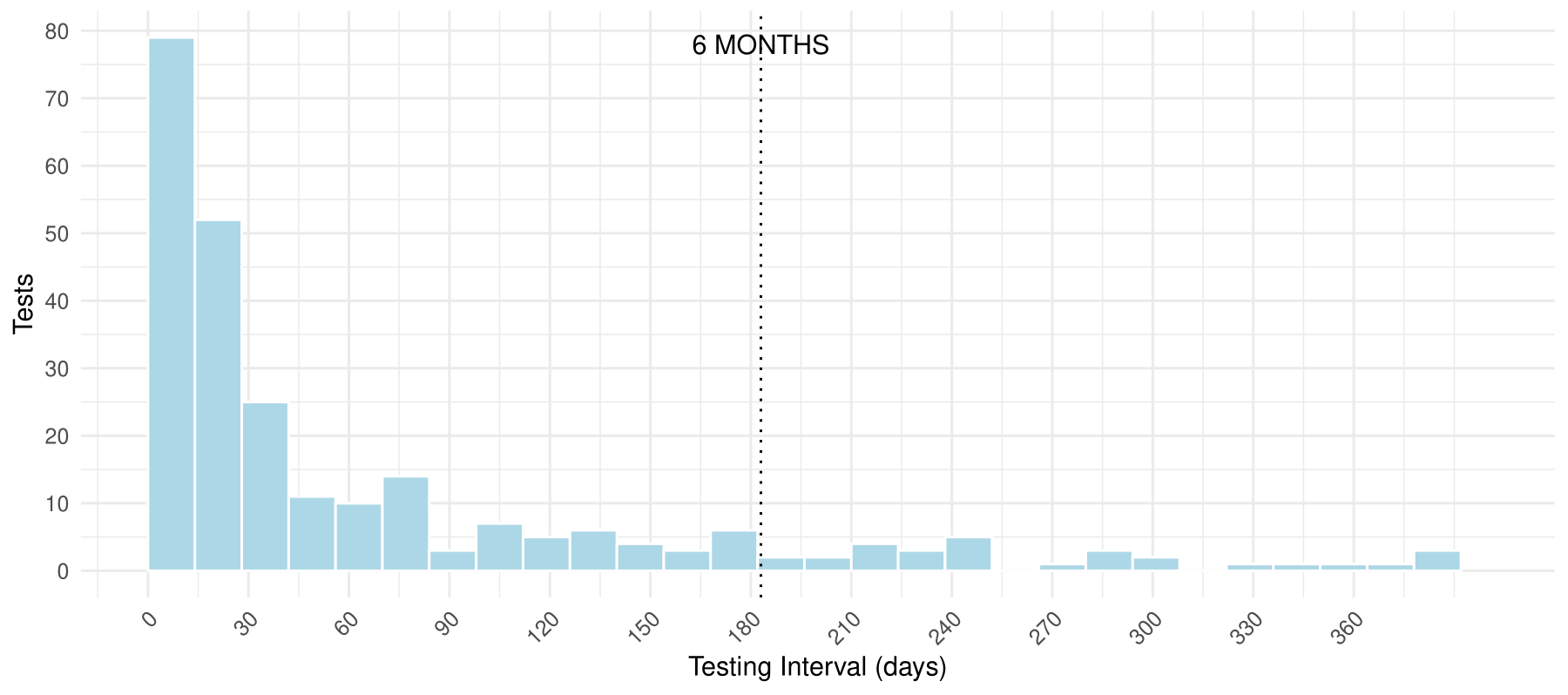

Supplement: Kaur et al. supplementary material [file S0899823X25103905sup001.tiff]
